# Competence of health professionals in problematizing and reflecting on the situations of people with chronic conditions*

**DOI:** 10.1590/1980-220X-REEUSP-2024-0314en

**Published:** 2025-05-12

**Authors:** Thaise Honorato de Souza, Saulo Fabio Ramos, Janaína da Silva Flôr, Joice Cristina Guesser, Natália Gonçalves, Vânia Marli Schubert Backes, Roberta Waterkemper, Monica Motta Lino

**Affiliations:** 1 Universidade Federal de Santa Catarina, Programa de Pós-graduação em Enfermagem, Florianópolis, SC, Brazil.

**Keywords:** Professional Competence, Comprehensive Health Care, Noncommunicable Diseases, Renal Insufficiency, Chronic, Educativo, Continuing

## Abstract

**Objective:**

To assess the impact of the Specialization Course in Care for People with Chronic Non-Communicable Diseases on health professionals’ work, focusing on the ability to problematize and reflect on situations of people with chronic conditions.

**Method:**

Ex-post facto evaluation based on the Experiential Learning Cycle, with emphasis on reflective observation. Conducted between May 2023 and June 2024, the research followed COREQ guidelines and included semi-structured interviews with 41 graduates, after ethical approval. Forty codes, 4 subcategories, and a central axis were identified. Funding: CNPq and FAPESC.

**Results:**

Reflective observation, according to the Theory of Experiential Learning, was fundamental to developing skills in problematizing and reflecting on situations and contexts experienced by people with chronic conditions.

**Conclusion:**

The study highlights the importance of reflective observation in the development of skills for the management of chronic conditions and the integration of Experiential Learning Theory in the training of health professionals to improve care.

## INTRODUCTION

David Kolb, in his Theory of Experiential Learning (TEL), highlights the importance of practical experiences in building skills and knowledge necessary for problem-solving, emphasizing that “knowledge is built from the transformation of experience”^(1)^.

Reflective Observation (RO), discussed in this article, constitutes one of the parts of the Kolb Cycle. It refers to problematization and expresses the ability to examine, investigate and measure or reflect critically on situations, facts, or contexts experienced in the territory by people with chronic conditions. The focus is on reflection and observation of lived experiences. It involves carefully observing problems and seeking to understand how events occur. Developing this competence enables professionals to review clinical cases, have group discussions, or write reflective journals^(1)^. Competence is the ability of a professional to perform a specific task, supported by appropriate training and education^(2)^.

Recent studies highlight the relevance of developing the ability to problematize and critically reflect on health practices. Critical thinking skills not only improve decision-making, but also increase the quality of patient care^(3)^. Furthermore, promoting critical reflection and problematization in the context of health education is fundamental for the training of more independent and efficient professionals. These competencies are relevant to face the challenges of an ever-changing healthcare environment and to promote evidence-based interventions.

Effective health practice requires the integration of knowledge, skills, and attitudes appropriate to the population’s realities and health needs. Efficient professional performance must be based on actions that involve autonomy, ethics, and the ability to diagnose and solve problems within the scope of healthcare and management^(4)^. The quality of health services depends on how professionals combine their values, knowledge, skills, and behaviors to respond to the needs of the community^(5)^.

This study is part of the doctoral dissertation in nursing, linked to the Macroproject “Evaluation of the post-professional qualification impact of workers, managers, and specialists in chronic non-communicable diseases”, and aims to evaluate the impact on the work process of health professionals who graduated from the “Specialization Course in Care for People with Chronic Non-Communicable Diseases (CNCD)”, focusing on the competence to problematize and reflect on situations and contexts experienced by people with chronic conditions, in the territory. The research is based on Kolb’s Experiential Learning Cycle, with an emphasis on the RO dimension.

## METHOD

### Design of Study

Ex-post-facto, qualitative impact assessment, in light of TEL, focusing on the RO of health professionals who completed a Specialization Course. The study used the instrument *Consolidated criteria for reporting qualitative research* (COREQ)^(6)^ to check all stages of the method.

### Local

This study was carried out in six municipalities in the State of Santa Catarina: Blumenau, Chapecó, Criciúma, Florianopolis, Joinville and Lages.

### Population

The “Specialization Course in Care for People with Chronic Non-Communicable Diseases,” promoted by UFSC between 2023 and 2024, qualified health professionals in Santa Catarina from the Brazilian Public Health System, using a hybrid approach and Kolb’s Experiential Learning Theory. With 360 hours, divided between the Integrative Axis, which addressed public policies and health promotion, and the Specialized Axis, focused on the management of chronic diseases such as diabetes and hypertension, the course encouraged the practical application of learning through intervention projects.

The research included a universe of 337 health professionals who graduated from the Specialization Course. The sample was intentionally stratified and distributed by center and different health professions, including 41 interviews with course graduates (12%), which theoretically saturated the theme.

### Data Collection

Data collection took place from June to September 2023 and included the following steps: (i) invitation and acceptance: sent via WhatsApp® to professionals who completed the Specialization Course; (ii) scheduling and conducting the interviews: with prior scheduling and being conducted virtually; (iii) conducting the interview carried out by the researcher: on the scheduled day, the access link was sent, followed by the reading of the research objectives, request for consent to conduct the interview and presentation of the semi-structured script addressing topics such as experiences during the course, changes in professional practices and application of knowledge in clinical practice. The interviews, with an average duration of 40 minutes, were performed and recorded via Google Meet^®^ and later transcribed by the researcher.

### Data Analysis and Treatment

Data were subjected to thematic content analysis^(7)^, which comprises three phases, in light of Kolb’s TEL: 1) pre-analysis, in which the material was organized, followed by 2) exploration, in which the information was aggregated into thematic categories and, finally, 3) treatment of the gross results and their interpretation, to then propose inferences, all of which carried out manually by the researcher. The 206 pages of material were analyzed during the months of June 2023 to June 2024. Forty codes emerged, resulting in 4 subcategories and a central analysis axis: the RO dimension of the Experiential Learning Cycle. OR, in the Course, refers to problematization and expresses the ability to examine, investigate, and measure or reflect critically on situations, facts, or contexts experienced in the territory by people with chronic conditions.

### Ethical Aspects

Article approved by the UFSC Research Ethics Committee, according to opinion No. 6.078.402. The research complies with Resolution 466 of December 12, 2012. To preserve the identity of the participants, pseudonyms were adopted according to their profession and sequential number, such as Fi1 (Physiotherapist 1).

## RESULTS

Forty-one (12%) healthcare professionals distributed across six centers within the State of Santa Catarina/SC were interviewed (Table 1).

**Table 1:** Characterization of study participants according to the hub, profession, and theme of the Undergraduate Thesis (*TCC*) – Florianópolis, SC, Brazil, 2024. (n = 41).

Hub	n	Occupation	TCC Theme*
Blumenau	5	04 Nurses 01 Pharmacist	Pharmaceutical assistance, cervical cancer, DM, SH
Chapecó	5	01 Social Worker 01 Dentist 02 Nurses 01 Pharmacist	DM, SH, obesity
Criciuma	11	01 Social Worker 05 Nurses 02 Pharmacists 01 Physiotherapist 01 Nutritionist 01 Psychologist	DM, SH, obesity, mental health, smoking
Florianopolis	6	05 Nurses 01 Nutritionist	DM, SH, obesity, mental health, smoking
Joinville	7	04 Nurses 01 Physiotherapist 01 Doctor 01 Nutritionist	DM, SH
Lages	7	01 Physical Educator 05 Nurses 01 Physiotherapist	Physical activity, depression, SH, obesity, smoking

The analysis of the transcribed interviews based on Kolb’s Experiential Learning Cycle resulted in the identification of 152 codes, which were organized into 16 subcategories and 4 categories, according to their similarities.

The Concrete Experience (CE) category highlighted elements related to the practical experience of participants, including participation and the initial impact of the course, the use of teaching materials and methods, interaction and support between professionals, as well as the challenges faced at the beginning of the training process. In the Reflective Observation (RO) category, critical reflections on professional practice, exchanges of feedback and suggestions for improvements were highlighted, in addition to the identification of changes observed in local contexts and strategic planning based on these observations.

The Abstract Conceptualization (AC) category addressed the systematization of learning, highlighting the theoretical development of participants, the application of learned concepts in practical contexts, the construction and testing of hypotheses and the integration of new knowledge with existing practices. Finally, the Active Experimentation (AE) category reflected the practical application of the knowledge acquired, encompassing the implementation and adaptation of strategies, the development and testing of interventions, the search for continuous improvements and practical solutions, in addition to the use of theoretical knowledge in concrete actions.

In this article, the category “Reflective Observation” will be highlighted, which emerged from the data in line with Kolb’s TEL framework.

The “Reflective Observation” category emerged as a central dimension in the development of skills of health professionals who finished the “Specialization Course in Care for People with Chronic Non-Communicable Diseases”. Based on the Experiential Learning Cycle, this competence, acquired in the Specialization Course and highlighted in this category, allowed professionals to critically reflect on the situations and contexts experienced by people with chronic conditions. By problematizing their practices, professionals were able to develop new care approaches and improve the quality of care.

Within this category, 40 codes and four subcategories emerged, called: Reflection on Practice, Feedback and Improvements, Observation of Changes, and Critical Review and Planning (Table 2).

**Table 2:** – Reflective Observation category, subcategories and codes – Florianópolis, SC, Brazil, 2024.

Subcategory	Codes
Reflection on Practice	Reflection on practice, Critical evaluation of the course, Personal reflection, Case analysis, Discussion of challenges, Review of prior knowledge, Comparison with previous experiences, Identification of gaps, Review of objectives, Reflection on errors, Reflection on feedback received, Reflection on difficulties, Reflection on learning, Analysis of interventions.
Feedback and improvements	Reflective feedback, Reflection on feedback received, Adjustments in professional practice, Discussion of experience, Planning for improvements, Self-assessment, Review of strategies, Review of processes, Review of results, Analysis of feedback, Review of materials, Review of the literature, Discussion of improvements, Planning of new approaches.
Observation of changes	Observation of changes, Success Identification, Results reviewing, Discussion of alternative approaches, Reflection on results, Observation of peers’ practices, Observation of effective practices, Observation of trends, Identification of good practices, Reflection on impact.
Critical review and planning	Review of strategies, Reflection on difficulties, Review of materials, Discussion of techniques, Review of processes, Discussion of innovations, Discussion of techniques, Discussion of cases, Discussion of experiences, Discussion of challenges.

### Reflection on Practice

Within the subcategory “Reflection on Practice”, graduates reported that the use of diaries was an important tool for bringing theory into practice in the municipality, allowing for in-depth reflection on their actions experienced in the context of CNCDs. For example, one professional commented: *Regarding the diaries, it is something that helps us bring it into the reality of the municipality, it makes us reflect on what we already do and what can be improved* (Fi3). This ongoing reflection allowed professionals to critically evaluate their practices and identify areas for improvement.

Critical evaluation of the course was also a significant aspect in this subcategory. Participants recognized the usefulness of the course in improving the management of people with CNCD, as mentioned: *The course helped, it was very useful in that part too* (Fa3). Personal reflections brought to light anxieties related to the limitations faced in professional practice, such as the lack of resources or specialized personnel. A nutritionist highlighted: *There’s no nutritionist, you know? There is one nutritionist per region, this nutritionist has to take care of four neighborhoods* (N2).

Case analysis and discussion of challenges in the course were highlighted as valuable tools for contextualizing theoretical knowledge in daily practice. *The exchange of experiences in the discussion forums helped to identify common problems and innovative solutions, highlighting the importance of critical observation and reflection in the continuous improvement of health practices. There was an interaction between colleagues* (...) *an exchange of experience* (Fi1).

The student values and reflects on the importance of reviewing the knowledge learned to improve their practice with people with CNCD: *For me, this knowledge gave me more security in relation to my professional and personal performance, I felt more confident when it came to guiding and accompanying. Brings more security* (...) *support* (E16).

### Feedback and Improvements

The “Feedback and Improvements” subcategory highlighted the importance of interaction in forums and the exchange of ideas as a way of enriching individual and collective reflection. A participant commented*: The exchange with other people, other experiences there to participate in the debates, in the forums, was also very interesting.* (E15PCr). This interaction allowed the comparison of practices and the incorporation of new ideas, promoting continuous and collaborative learning.

The feedback received from tutors and colleagues was considered valuable for self-assessment and for adjustments in professional practice. The teachers’ guidance and face-to-face meetings were mentioned as important moments for critical reflection: *These meetings, I always consider very enriching.* (...) *to learn and exchange* (E11).

Discussions of experiences with other professionals allowed a detailed analysis of practices and the identification of areas that needed improvement, as can be seen in this report: *I think the forum is very important because we are so focused on our region, on our city, that we don’t look at others. And sometimes what works in one city might work for me* (E19).

Planning for improvements was facilitated by the use of diaries and ongoing reflection on current practices: *Regarding the diaries, it is something that helps us bring it into the reality of the municipality, it makes us reflect on what we already do and what can be improved* (Fi3).

Self-assessment has emerged as an essential component for professional development, allowing graduates to identify their strengths and weaknesses and constantly seek to improve their skills. *Therefore, this specialization was extremely rewarding, because it contributed to my learning and allowed me to reinforce this learning* (E25).

### Change Observation

The “Observation of Changes” subcategory highlighted the professionals’ ability to analyze their practical experiences, identify areas for improvement and consolidate learning. *I learned a lot during this year of the course, which also helped me to better manage patients and explain certain things to them. In this matter of talking to the patient, explaining helped me a lot* (Fa3). Sharing experiences in forums and face-to-face meetings provided a space for collective reflection, essential to broaden understanding of care practices: *The face-to-face meeting was also very enriching due to the exchange of experiences, as it was a postgraduate course that had many people from other specialties* (E17).

Reviewing results and discussing alternative approaches allowed practitioners to evaluate different perspectives and adapt their practices as needed. Reflecting on the results achieved was important to validate the learning process and implement significant changes in care practices: *It helped me a lot to understand some things, patient management, treating the different types of patients we have* (Fa3).

### Critical Review and Planning

In the subcategory “Critical Review and Planning”, graduates emphasized the importance of reviewing strategies and reflecting on difficulties faced in practice. RO allowed professionals to analyze their experiences, identify areas for improvement and consolidate learning. One participant highlighted: *Nowadays we end up focusing on the patient when the disease has already taken hold and I believe that prevention would be the best way* (N2).

Review of materials and discussiion of techniques were mentioned as effective methods for deepening knowledge and improving professional practices. Discussion forums, synchronous classes, and face-to-face meetings encouraged professionals to share ideas and solutions, promoting a collaborative learning environment: *The exchange with other people, other experiences there to participate in the debates, in the forums, was also very interesting* (E15).

Process review was identified as an important step to consolidate learning and ensure continuous improvement of health practices: *Everything we learn somehow changes our vision, our outlook on everyday things, especially in health.* (E15). Reflective observation, therefore, not only promoted the development of critical skills, but also facilitated adaptation and innovation in health care practices.

## DISCUSSION

The study demonstrates that professional training in health, in the course under study, based on Kolb’s TEL, enhances the development of critical reflective skills through strategies such as field diaries, discussion forums, case analysis, and collaborative feedback, allowing professionals to connect theory and practice, identify their limitations, develop more adaptive and innovative practices, and promote a more holistic and contextualized approach to the care of chronic conditions, overcoming the traditional prescriptive and curative model, by stimulating a professional practice based on continuous reflection, the exchange of experiences, and the constant redefinition of knowledge.

### Reflection on Practice

Health professionals reported that tools such as diaries and case discussions allowed them to connect theory and practice, promoting ongoing critical review. This result reinforces the importance of reflection for transformative practice, as described in Kolb’s TEL. Similar studies corroborate the effectiveness of reflective strategies in improving clinical care, which allows professionals to challenge their own assumptions and biases, leading to transformative learning and the development of more effective practices^(8)^.

Kolb highlights the importance of considering multiple perspectives during reflection, meaning that experts should look at the experience from different angles and consider different interpretations and meanings^(1)^. This approach enables professionals to develop a deeper understanding of best care practices, essential for the effective management of chronic conditions.

Interaction in forums and the exchange of ideas were important for continuous learning, as reported by graduates. This type of collaborative learning is recognized in the literature as beneficial for professional development. Communities of practice, such as discussion forums, provide a space for professionals to share experiences, solve common problems, and develop new skills^(9,10)^.

RO, as described in Kolb’s TEL, is fundamental for the development of competences to problematize and reflect on situations and contexts experienced by people with chronic conditions. Professionals who completed the specialization course in CNCDs highlighted the importance of critical reflection on their daily practices and how this reflection is facilitated by educational tools, such as field diaries, discussion forums, and synchronous meetings. Reflective practice enables professionals to identify areas for improvement and adapt their approaches to better meet the needs of people with chronic conditions^(11)^.

The activity of permanent education in health, through the problematization of everyday situations in the face of chronic conditions and group methodologies, motivates health professionals. Faced with the problem, they stop, examine, reflect, relate their history, and begin to re-signify their discoveries. Continuing education activities aligned with the practice of health services, based on group methodologies, enable freedom and autonomy in making choices and decisions. It is necessary to innovate for active and problem-solving educational approaches^(11)^.

Professionals from the specialization course reported several difficulties and limitations in the management and assistance of chronic conditions, such as the lack of human resources and the need to adapt to local conditions. The speech “There are no nutritionists, you know? There is one nutritionist per region, this nutritionist has to take care of four neighborhoods (N2)” exemplifies the work overload and lack of resources. These challenges directly impact the quality of care provided and the ability to implement preventive strategies^(12)^.

Prevention of chronic conditions is recognized as an essential strategy, but professionals point out that the current focus is predominantly on curative care. The speech “Nowadays we end up focusing on the patient when the disease has already taken hold and I believe that prevention would be the best way (N2)” highlights this concern. Moreover, continuous review of knowledge is seen as a strategy to improve professional practice, as illustrated in the excerpt “the readings helped us to be able to write, respond, all of that” (E17).

There is a predominance of the prescriptive care model, focused on the disease and with little involvement of teams in comprehensive health care and user empowerment. Improving the health of people with chronic conditions requires transforming systems that are essentially reactive (which respond primarily when a person is ill) into proactive systems that focus on keeping the person as healthy as possible^(13,14)^.

Chronic conditions require ongoing care and ongoing education for both healthcare staff and patients. This process is essential to clarify doubts about the ongoing treatment and to address issues that may arise during adherence to treatment. The exchange of information between the patient and the healthcare unit is essential to build a relationship of trust and effectiveness. The continuity of this care, guided by guidelines and protocols established by the healthcare team, results in more accurate diagnoses and treatments. Furthermore, it ensures adequate guidance throughout the assistance, providing personalized and effective care for each individual^(14,15,16)^.

Managing learning and knowledge in healthcare institutions represents a significant challenge for professionals in the field. Developing effective strategies for creating, maintaining, and sharing information and knowledge is essential. These strategies allow healthcare professionals to quickly access the alternatives needed to make informed and effective decisions^(17)^.

Reflective feedback, obtained through interaction in forums and face-to-face meetings, contributes to the improvement of professional practices, as can be seen in the statements: The exchange with other people, other experiences there to participate in the debates, in the forums, was also very interesting (E15). These meetings, I always consider very enriching to learn and exchange (E11). This highlights the importance of exchanging experiences and ideas between professionals from different municipalities and contexts. These moments of exchange and feedback contribute to a richer and more detailed analysis of experiences, helping professionals to identify areas that can be improved and to implement effective changes. Furthermore, a review of learning outcomes and the evaluation of alternative approaches to problem-solving assist specialists in applying the acquired knowledge to develop effective strategies for managing people with chronic conditions in the territory^(17)^.

### Change Observation

The ability to observe and reflect on changes in practices highlighted the role of the course in professional adaptation to dynamic health scenarios. Critical reflection on the results achieved demonstrates the positive impact of reflective learning on the consolidation of evidence-based practices. Recent studies claim that education based on critical reflection improves professionals’ ability to respond to complex situations in the workplace^(3)^.

Personal reflection, which brought to light anxieties related to limitations faced in practice, is also a common theme in the literature, where lack of resources and work overload are often cited as significant challenges^(18,19)^. Case analysis and discussion of challenges in the course were highlighted as valuable tools for contextualizing theoretical knowledge in daily practice.

The exchange of experiences in the discussion forums helped to identify common problems and innovative solutions, highlighting the importance of critical observation and reflection in the continuous improvement of health practices. This practice of “reflection-in-action” is essential for the continuous improvement of health practices, as it allows professionals to adapt their approaches based on experiences and feedback^(20)^.

The ability of professionals to analyze their practical experiences and identify areas for improvement was an aspect highlighted by a subcategory. Reflecting on past experiences is essential for continuous learning and adaptation of practices^(21,22)^. The literature also suggests that the ability to observe and reflect on changes is important for implementing improvements in health practices^(23)^.

### Feedback and Improvements

Feedback was highlighted as an essential element for self-assessment and adjustments in practice. Interaction in forums and face-to-face meetings fostered enriching discussions, allowing professionals to identify gaps and implement innovative strategies. This finding is in line with the literature, which positions feedback as a powerful tool for continuous improvement.

The feedback received from tutors and colleagues was considered valuable for adjustments in professional practice. The literature highlights that constructive feedback is a critical component of reflective learning, as it allows professionals to identify areas for improvement and develop strategies to address these areas^(24)^. Feedback is one of the most powerful tools for continuous improvement as it provides valuable insights into current practices and potential improvements^(25)^.

Improvement planning and self-assessment emerged as essential components of ongoing professional development. Recent studies claim that self-assessment helps professionals recognize their own skills and limitations, promoting a cycle of continuous improvement^(26)^. Self-assessment is fundamental to developing a reflective practice, where professionals are constantly evaluating and adjusting their approaches to best meet patients’ needs^(21)^.

Sharing experiences in forums and face-to-face meetings allowed for collective reflection, which is essential for expanding understanding of care practices. This finding is corroborated by studies showing that collaboration between professionals from different areas of expertise enriches practice and promotes a more holistic approach to care. Learning in communities of practice provides a rich exchange of knowledge and experiences that is fundamental to continuous improvement^(27)^.

### Critical Review and Planning

The subcategory highlights the importance of reviewing strategies and planning actions based on practical challenges. Collaborative tools, such as forums and face-to-face discussions, facilitated the deepening of knowledge and innovation in care practices.

In the subcategory “Critical Review and Planning”, graduates emphasized the importance of reviewing strategies and reflecting on difficulties faced in practice. The literature suggests that critically reviewing strategies allows practitioners to adjust their approaches based on evidence and best practices^(28)^. This practice is essential to ensure that the care provided is always the most effective and based on the best evidence available.

Review of materials and discussion of techniques were effective methods for deepening knowledge and improving professional practices. Continuous learning and review of teaching materials are essential to stay up to date with best practices and the latest evidence^(21)^. Critical review and discussion of techniques are fundamental to innovation and improvement of health practices^(23)^.

Process review was identified as an important step to consolidate learning and ensure continuous improvement of healthcare practices. This finding is supported by studies showing that regular process review allows professionals to identify inefficiencies and implement improvements^(1)^. Furthermore, systematic review of processes is essential for implementing evidence-based practices and adapting to changing patient needs^(29)^.

### Limitations and Future Perspectives

This study has limitations related to the generalization of results, due to the exclusive focus on graduates of a specific course. Future research can explore the impact of reflective training longitudinally and compare different pedagogical approaches, deepening the understanding of reflective competencies in health.

Although this study provided valuable results on the development of reflective and critical skills by health professionals, it is important to recognize some limitations. First, the sample consisted exclusively of graduates of a specific specialization course in chronic non-communicable diseases, which may limit the generalization of the results to other areas of specialization or educational contexts. Second, the qualitative nature of the study, based on semi-structured interviews, may be subject to biases in self-reporting and data interpretation, even though these were validated by the research participants themselves.

Furthermore, this study focused predominantly on the Reflective Observation dimension of Kolb’s Experiential Learning Cycle, failing to explore other dimensions of the cycle, such as concrete experience, active experimentation, and abstract conceptualization, which are also important for the complete development of professional competencies.

Finally, future research could longitudinally explore the impact of competencies developed over time, assessing how reflective training influences professional practice and quality of care in the long term. Comparative studies between different training programs and pedagogical methods would also be valuable to identify the most effective approaches for developing critical and reflective skills among health professionals.

## CONCLUSION

This study highlights the importance of problematization, critical reflection, and RO in the training of health professionals, particularly in the context of CNCD management, through methodologies that encourage in-depth analysis of daily practices, such as field diaries, discussion forums, and synchronous meetings. It was possible to identify significant improvements in care approaches. These educational tools not only facilitate collaborative learning, but also promote a culture of self-assessment and continuous improvement among professionals.

Continuing health education, through the problematization of everyday situations and group methodologies, motivates health professionals to reflect on their practices and to re-signify their discoveries. The exchange of experiences and ideas between professionals from different contexts contributes to a richer and more detailed analysis of experiences, promoting the implementation of effective changes.

The integration of Kolb’s TEL into the specialization curriculum has proven to be an effective model for developing critical and reflective competences. This approach opens doors for health professionals to adapt their practices to the specific realities of their territories, improving the quality of care and responding more precisely to individual and collective needs.

However, the study also reveals persistent challenges, such as work overload and scarcity of resources, which impact the implementation of preventive strategies and comprehensive health care. These obstacles highlight the need for more robust public policies and continued investment in training and support for health professionals.
