# Supplementary material for: Competence of health professionals in problematizing and reflecting on the situations of people with chronic conditions
Source: Rev Esc Enferm USP. 2025 May 12;59:e20240314. doi: 10.1590/1980-220X-REEUSP-2024-0314en (PMC12080369; doi:10.1590/1980-220X-REEUSP-2024-0314en)
Supplement: Translation

# Translation: Competência de profissionais da saúde em problematizar e refletir sobre situações de pessoas com condições crônicas*

**DOI:** 10.1590/1980-220X-REEUSP-2024-0314pt

**Authors:** Thaise Honorato de Souza, Saulo Fabio Ramos, Janaína da Silva Flôr, Joice Cristina Guesser, Natália Gonçalves, Vânia Marli Schubert Backes, Roberta Waterkemper, Monica Motta Lino

**Affiliations:** 1 Universidade Federal de Santa Catarina, Programa de Pós-graduação em Enfermagem, Florianópolis, SC, Brasil.

**Keywords:** Competência Profissional, Assistência Integral à Saúde, Doenças não transmissíveis, Insuficiência Renal Crônica, Educação Continuada

## Abstract

**Objetivo:**

Avaliar o impacto do Curso de Especialização em Atenção às Pessoas com Doenças Crônicas Não-Transmissíveis no trabalho dos profissionais de saúde, focando na competência de problematizar e refletir sobre situações de pessoas com condições crônicas.

**Método:**

Avaliação ex-post facto baseada no Ciclo de Aprendizagem Experiencial, com ênfase na observação reflexiva. Realizada entre maio de 2023 e junho de 2024, a pesquisa seguiu as diretrizes do COREQ e incluiu entrevistas semiestruturadas com 41 egressos, após aprovação ética. Foram identificados 40 códigos, 4 subcategorias e um eixo central. Financiamento: CNPq e FAPESC.

**Resultados:**

A observação reflexiva, conforme a Teoria da Aprendizagem Experiencial, foi fundamental para desenvolver competências em problematizar e refletir sobre situações e contextos vivenciados por pessoas com condições crônicas.

**Conclusão:**

O estudo ressalta a importância da observação reflexiva na formação de competências para o manejo de condições crônicas e a integração da Teoria da Aprendizagem Experiencial na formação de profissionais de saúde para melhorar o atendimento.

## INTRODUÇÃO

David Kolb, em sua Teoria da Aprendizagem Experiencial (TAE), destaca a importância das experiências práticas na construção de habilidades e conhecimentos necessários para a resolução de problemas, ressaltando que o “conhecimento é construído a partir da transformação da experiência”^(1)^.

A Observação Reflexiva (OR), discutida neste artigo, constitui-se uma das partes do Ciclo de Kolb. Refere-se à problematização e exprime a capacidade de examinar, investigar e mensurar ou refletir de forma crítica sobre situações, fatos ou contextos vivenciados no território, por pessoas com condições crônicas. O foco é na reflexão e observação das experiências vividas. Envolve a observação cuidadosa dos problemas e a busca por compreender como os eventos ocorrem. Desenvolver essa competência possibilita aos profissionais a revisão de casos clínicos, discussões em grupo ou a escrita de diários reflexivos^(1)^. Competência é a capacidade de um profissional executar uma tarefa específica, respaldado pelo treinamento e educação adequados^(2)^.

Estudos recentes destacam a relevância do desenvolvimento da competência em problematizar e refletir criticamente sobre práticas de saúde. A capacidade de reflexão crítica não apenas melhora a tomada de decisões, mas também aumenta a qualidade do atendimento ao paciente^(3)^. Além disso, a promoção da reflexão crítica e da problematização no contexto da educação em saúde é fundamental para a formação de profissionais mais independentes e eficientes. Essas competências são relevantes para enfrentar os desafios de um ambiente de saúde em constante mudança e para promover intervenções baseadas em evidências.

A prática efetiva na saúde requer a integração de conhecimentos, habilidades e atitudes adequadas às realidades e necessidades de saúde da população. A atuação profissional eficiente deve ser baseada em ações que envolvem autonomia, ética e a capacidade de diagnosticar e solucionar problemas no âmbito da atenção e gestão do cuidado em saúde^(4)^. A qualidade dos serviços de saúde depende de como os profissionais combinam seus valores, conhecimentos, competências e comportamentos para responder às necessidades da comunidade^(5)^.

Este estudo integra a tese de doutorado em enfermagem, vinculada ao Macroprojeto “Avaliação do impacto pós-qualificação profissional de trabalhadores, gestores e especialistas em doenças crônicas não transmissíveis”, e visa avaliar o impacto no processo de trabalho dos profissionais de saúde egressos do “Curso de Especialização em Atenção às Pessoas com Doenças Crônicas Não-Transmissíveis (CCNT)”, focando na competência de problematizar e refletir sobre situações e contextos vivenciados por pessoas com condições crônicas, no território. A pesquisa é fundamentada no Ciclo de Aprendizagem Experiencial de Kolb, com ênfase na dimensão de OR.

## MÉTODO

### Desenho do Estudo

Avaliação de impacto do tipo ex-post-facto, qualitativa, à luz da TAE, com enfoque na OR de profissionais da saúde egressos de um Curso de Especialização. O estudo utilizou o instrumento *Consolidated criteria for reporting qualitative research* (COREQ)^(6)^ para conferência de todas as etapas do método.

### Local

O presente estudo foi realizado em seis municípios do Estado de Santa Catarina: Blumenau, Chapecó, Criciúma, Florianópolis, Joinville e Lages.

### População

O “Curso de Especialização em Atenção às Pessoas com Doenças Crônicas Não-Transmissíveis,” promovido pela UFSC entre 2023 e 2024, qualificou profissionais da saúde do SUS em Santa Catarina, utilizando uma abordagem híbrida e a Teoria de Aprendizagem Experiencial de Kolb. Com 360 horas, divididas entre o Eixo Integrador, que abordou políticas públicas e promoção da saúde, e o Eixo Especializado, focado no manejo de doenças crônicas como diabetes e hipertensão, o curso incentivou a aplicação prática do aprendizado por meio de projetos de intervenção.

A pesquisa incluiu um universo de 337 profissionais da saúde egressos do Curso de Especialização. A amostra foi estratificada intencionalmente e distribuída por polo e diferentes profissões da saúde, contemplando 41 entrevistas com egressos do curso (12%), que saturaram teoricamente a temática.

### Coleta de Dados

A coleta de dados ocorreu de junho a setembro de 2023 e incluiu as seguintes etapas: (i) convite e aceite: enviado via WhatsApp® aos profissionais egressos do Curso de Especialização; (ii) agendamento e realização das entrevistas: com agendamento prévio e sendo realizadas virtualmente; (iii) condução da entrevista realizada pelo pesquisador: no dia agendado, era enviado o link de acesso, seguido pela leitura dos objetivos da pesquisa, solicitação de consentimento para a realização da entrevista e apresentação do roteiro semiestruturado abordando temas como experiências durante o curso, mudanças nas práticas profissionais e aplicação dos conhecimentos na prática clínica. As entrevistas, com duração média de 40 minutos, foram realizadas e gravadas pelo Google Meet® e posteriormente transcritas.

### Análise e Tratamento dos Dados

Os dados foram submetidos à análise de conteúdo temática^(7)^, que compreende três fases, à luz da TAE de Kolb: 1) pré-análise, em que o material foi organizado, seguida da 2) exploração, em que as informações foram agregadas em categorias temáticas e, por último, 3) tratamento dos resultados brutos e sua interpretação, para, então, propor inferências, tudo isso realizado manualmente pelo pesquisador. As 206 páginas de material foram analisadas durante os meses de junho de 2023 a junho de 2024. Emergiram 40 códigos, resultando em 04 subcategorias e um eixo de análise central: a dimensão OR, do Ciclo de Aprendizagem Experiencial. A OR, no Curso, refere-se à problematização e exprime a capacidade de analisar, investigar, mensurar, problematizar e refletir de forma crítica sobre situações, fatos ou contextos vivenciados no território por pessoas com condições crônicas.

### Aspectos Éticos

Artigo aprovado pelo Comitê de Ética em Pesquisa da UFSC, conforme parecer N6 .078.402. A pesquisa está em conformidade com a Resolução 466, de 12 de dezembro de 2012. Para preservar a identidade dos participantes, adotaram-se pseudônimos conforme profissão e número sequencial, como o exemplo Fi1 (Fisioterapeuta 1).

## RESULTADOS

Foram entrevistados 41 (12%) profissionais da área da saúde distribuídos em seis polos dentro do Estado de Santa Catarina/SC (Tabela 1).

**Tabela 1:** Caracterização dos participantes do estudo segundo polo, profissão e tema do Trabalho de Conclusão de Curso – Florianópolis, SC, Brasil, 2024 (n = 41).

Polo	n	Profissão	Tema do TCC*
Blumenau	5	04 Enfermeiros 01 Farmacêutico	Assistência farmacêutica, câncer de colo de útero, DM, HAS
Chapecó	5	01 Assistente Social 01 Dentista 02 Enfermeiros 01 Farmacêutico	DM, HAS, obesidade
Criciúma	11	01 Assistente Social 05 Enfermeiros 02 Farmacêuticos 01 Fisioterapeuta 01 Nutricionista 01 Psicólogo	DM, HAS, obesidade, saúde mental, tabagismo
Florianópolis	6	05 Enfermeiros 01 Nutricionista	DM, HAS, obesidade, saúde mental, tabagismo
Joinville	7	04 Enfermeiros 01 Fisioterapeuta 01 Médico 01 Nutricionista	DM, HAS
Lages	7	01 Educador Físico 05 Enfermeiros 01 Fisioterapeuta	Atividade física, depressão, HAS, obesidade, tabagismo

A análise das entrevistas transcritas com base no Ciclo de Aprendizagem Experiencial de Kolb resultou na identificação de 152 códigos, que foram organizados em 16 subcategorias e 4 categorias, conforme suas semelhanças.

A categoria Experiência Concreta (EC) destacou elementos relacionados à vivência prática dos participantes, incluindo a participação e o impacto inicial do curso, a utilização de materiais e métodos didáticos, a interação e o suporte entre os profissionais, bem como os desafios enfrentados no início do processo formativo. Na categoria Observação Reflexiva (OR), foram evidenciadas reflexões críticas sobre a prática profissional, trocas de feedback e sugestões de melhorias, além da identificação de mudanças observadas nos contextos locais e do planejamento estratégico baseado nessas observações.

A categoria Conceitualização Abstrata (CA) abordou a sistematização do aprendizado, evidenciando o desenvolvimento teórico dos participantes, a aplicação de conceitos aprendidos em contextos práticos, a construção e o teste de hipóteses e a integração de novos conhecimentos com as práticas existentes. Por fim, a categoria Experimentação Ativa (EA) refletiu a aplicação prática dos conhecimentos adquiridos, englobando a implementação e adaptação de estratégias, o desenvolvimento e teste de intervenções, a busca por melhorias contínuas e soluções práticas, além da utilização de conhecimento teórico em ações concretas.

Neste artigo, será destacada a categoria “Observação Reflexiva”, que emergiu dos dados em consonância com o referencial TAE de Kolb.

A categoria “Observação Reflexiva” emergiu como uma dimensão central no desenvolvimento de competências dos profissionais de saúde egressos do “Curso de Especialização em Atenção às Pessoas com Doenças Crônicas Não-Transmissíveis”. Baseando-se no Ciclo de Aprendizagem Experiencial, essa competência, adquirida no Curso de Especialização e sublinhada nesta categoria, permitiu que os profissionais refletissem criticamente sobre as situações e contextos vivenciados por pessoas com condições crônicas. Ao problematizar suas práticas, os profissionais puderam desenvolver novas abordagens de cuidado e aprimorar a qualidade do atendimento.

Dentro desta categoria, emergiram 40 códigos e quatro subcategorias, denominadas: Reflexão sobre a Prática, Feedback e Melhorias, Observação de Mudanças, e Revisão Crítica e Planejamento (Tabela 2).

**Tabela 2:** Categoria Observação Reflexiva, subcategorias e códigos – Florianópolis, SC, Brasil, 2024.

Subcategoria	Códigos
Reflexão sobre a Prática	Reflexão sobre a prática, Avaliação crítica do curso, Reflexão pessoal, Análise de casos, Discussão de desafios, Revisão de conhecimento prévio, Comparação com experiências anteriores, Identificação de lacunas, Revisão de objetivos, Reflexão sobre erros, Reflexão sobre feedback recebido, Reflexão sobre dificuldades, Reflexão sobre aprendizagem, Análise de intervenções.
Feedback e melhorias	Feedback reflexivo, Reflexão sobre feedback recebido, Ajustes na prática profissional, Discussão de experiência, Planejamento de melhorias, Autoavaliação, Revisão de estratégias, Revisão de processos, Revisão de resultados, Análise de feedback, Revisão de materiais, Revisão de literatura, Discussão de melhorias, Planejamento de novas abordagens.
Observação de mudanças	Observação de mudanças, Identificação de sucesso, Revisão de resultados, discussão de abordagem alternativas, Reflexão sobre resultados, Observação das práticas dos colegas, Observação de práticas eficazes, Observação de tendências, Identificação de boas práticas, Reflexão sobre impacto.
Revisão crítica e planejamento	Revisão de estratégias, Reflexão sobre dificuldades, Revisão de materiais, Discussão de técnicas, Revisão de processos, Discussão de inovações, Discussão de técnicas, Discussão de casos, Discussão de experiências, Discussão de desafios.

### Reflexão Sobre a Prática

Dentro da subcategoria “Reflexão sobre a Prática”, os egressos relataram que a utilização de diários foi uma ferramenta importante para trazer a teoria para a prática no município, permitindo uma reflexão profunda sobre suas ações vivenciadas no contexto das CCNT. Por exemplo, um profissional comentou: *Em relação aos diários, é algo que ajuda a gente trazer para a realidade do município, faz a gente fazer uma reflexão daquilo que a gente já pratica e daquilo que pode ser aprimorado* (Fi3). Essa reflexão contínua permitiu aos profissionais avaliarem criticamente suas práticas e identificar áreas de melhoria.

A avaliação crítica do curso também foi um aspecto significativo nesta subcategoria. Os participantes reconheceram a utilidade do curso na melhoria do manejo de pessoas com CCNT, como mencionado: *O curso ajudou, foi muito útil nessa parte também* (Fa3). Reflexões pessoais trouxeram à tona angústias relacionadas às limitações enfrentadas na prática profissional, como a falta de recursos ou de pessoal especializado. Um nutricionista destacou: *Não tem nutricionista, sabe? Tem uma nutricionista por região, essa nutricionista tem que cuidar de quatro bairros* (N2).

A análise de casos e a discussão de desafios no curso foram apontadas como ferramentas valiosas para contextualizar o conhecimento teórico na prática diária. *A troca de experiências nos fóruns de discussão ajudou a identificar problemas comuns e soluções inovadoras, evidenciando a importância da observação crítica e da reflexão na melhoria contínua das práticas de saúde. Houve uma interação entre os colegas* (...) *uma troca de experiência* (Fi1).

O aluno valoriza, reflete a importância de rever o conhecimento aprendido para melhorar a sua prática junto às pessoas com CCNT: *O conhecimento para mim trouxe mais segurança em relação a minha atuação profissional e pessoal, me senti mais segura na hora de orientar, de acompanhar. Traz mais segurança* (...) *suporte* (E16).

### Feedback e Melhorias

A subcategoria “Feedback e Melhorias” destacou a importância da interação nos fóruns e das trocas de ideias como forma de enriquecer a reflexão individual e coletiva. Um participante comentou*: A troca também com outras pessoas, outras experiências ali para participar dos debates, dos fóruns, foi bem interessante* (E15PCr). Essa interação permitiu a comparação de práticas e a incorporação de novas ideias, promovendo um aprendizado contínuo e colaborativo.

O feedback recebido dos tutores e colegas foi considerado valioso para a autoavaliação e para ajustes na prática profissional. A condução das professoras e os encontros presenciais foram mencionados como momentos importantes para a reflexão crítica: *Esses encontros, eu sempre acho que são muito ricos* (...) *para aprender e trocar* (E11).

As discussões de experiências com outros profissionais possibilitaram uma análise detalhada das práticas e a identificação de áreas que necessitavam de melhorias, como se pode verificar neste relato: *O fórum eu acho muito importante porque como a gente é tão centrado na nossa região, na nossa cidade, que a gente não observa outras. E às vezes o que funciona em uma cidade pode funcionar para mim* (E19).

O planejamento de melhorias foi facilitado pelo uso de diários e pela reflexão contínua sobre as práticas atuais: *Em relação aos diários, é algo que ajuda a gente trazer para a realidade do município, faz a gente fazer uma reflexão daquilo que a gente já pratica e daquilo que pode ser aprimorado* (Fi3).

A autoavaliação emergiu como um componente essencial para o desenvolvimento profissional, permitindo aos egressos identificarem suas fortalezas e fraquezas e buscar constantemente a melhoria de suas competências. *Então essa especialização foi extremamente gratificante, porque ela contribuiu para o meu aprendizado e para que eu pudesse reforçar esse aprendizado* (E25).

### Observação de Mudanças

A subcategoria “Observação de Mudanças” destacou a capacidade dos profissionais em analisar suas experiências práticas, identificar áreas de melhoria e consolidar o aprendizado. *Aprendi bastante coisa nesse ano de curso, que me ajudou também a ter um manejo com o paciente melhor, explicar algumas coisas para eles. Nessa questão de conversa com o paciente, explicar, me ajudou bastante* (Fa3). A partilha de experiências nos fóruns e encontros presenciais proporcionou um espaço para reflexão coletiva, essencial para ampliar o entendimento sobre as práticas de cuidado: *O encontro presencial também foi bem rico pela troca de experiências, por ser uma pós que tinha muita gente de outras especialidades* (E17).

A revisão de resultados e a discussão de abordagens alternativas permitiram aos profissionais avaliarem diferentes perspectivas e adaptar suas práticas conforme necessário. A reflexão sobre os resultados alcançados foi importante para validar o processo de aprendizado e implementar mudanças significativas nas práticas de cuidado: *Ajudou-me muito a compreender algumas coisas, o manejo com o paciente, atender os tipos diferentes de pacientes que a gente tem* (Fa3).

### Revisão Crítica e Planejamento

Na subcategoria “Revisão Crítica e Planejamento”, os egressos enfatizaram a importância da revisão de estratégias e da reflexão sobre dificuldades enfrentadas na prática. A OR permitiu que os profissionais analisassem suas experiências, identificassem áreas de melhoria e consolidassem o aprendizado. Um participante destacou: *Hoje em dia a gente acaba focando no paciente quando a doença já está instalada e eu acredito que a prevenção seria o melhor caminho* (N2).

A revisão de materiais e a discussão de técnicas foram mencionadas como métodos eficazes para aprofundar o conhecimento e aprimorar as práticas profissionais. Os fóruns de discussão, as aulas síncronas e os encontros presenciais incentivaram os profissionais a compartilharem ideias e soluções, promovendo um ambiente de aprendizado colaborativo: *A troca também com outras pessoas, outras experiências ali para participar dos debates, dos fóruns, foi bem interessante* (E15).

A revisão de processos foi identificada como uma etapa importante para consolidar o aprendizado e garantir a melhoria contínua das práticas de saúde: *Tudo que a gente aprende de alguma forma modifica a nossa visão, o nosso olhar perante as coisas do dia a dia, principalmente na saúde* (E15). A observação reflexiva, portanto, não apenas promoveu o desenvolvimento de competências críticas, mas também facilitou a adaptação e a inovação nas práticas de cuidado em saúde.

## DISCUSSÃO

O estudo demonstra que a formação profissional em saúde, do curso em estudo, fundamentada na TAE de Kolb, potencializa o desenvolvimento de competências reflexivas críticas por meio de estratégias como diários de campo, fóruns de discussão, análise de casos e feedback colaborativo, permitindo que os profissionais conectem teoria e prática, identifiquem suas limitações, desenvolvam práticas mais adaptativas e inovadoras, e promovam uma abordagem mais holística e contextualizada no cuidado às condições crônicas, superando o modelo tradicional prescritivo e curativista, ao estimular uma prática profissional baseada na reflexão contínua, na troca de experiências e na constante ressignificação do conhecimento.

### Reflexão Sobre a Prática

Os profissionais de saúde relataram que ferramentas como diários e discussões de casos permitiram conectar teoria e prática, promovendo uma revisão crítica contínua. Esse resultado reforça a importância da reflexão para a prática transformadora, conforme descrito na TAE de Kolb. Estudos semelhantes corroboram a eficácia de estratégias reflexivas no aprimoramento do atendimento clínico, que permite que os profissionais desafiem suas próprias suposições e preconceitos, levando a um aprendizado transformador e ao desenvolvimento de práticas mais eficazes^(8)^.

Kolb destaca a importância de considerar múltiplas perspectivas durante a reflexão, o que significa que os especialistas devem olhar para a experiência de diferentes ângulos e considerar diversas interpretações e significados^(1)^. Esta abordagem permite que os profissionais desenvolvam uma compreensão mais profunda das melhores práticas de cuidado, essencial para a gestão eficaz de condições crônicas.

A interação nos fóruns e a troca de ideias foram importantes para o aprendizado contínuo, conforme relatado pelos egressos. Esse tipo de aprendizado colaborativo é reconhecido na literatura como benéfico para o desenvolvimento profissional. Comunidades de prática, como fóruns de discussão, proporcionam um espaço para os profissionais compartilharem experiências, resolverem problemas comuns e desenvolverem novas competências^(9,10)^.

A OR, conforme descrita na TAE de Kolb, é fundamental para o desenvolvimento de competências em problematizar e refletir sobre situações e contextos vivenciados por pessoas com condições crônicas. Os profissionais egressos do curso de especialização em DCNT destacaram a importância da reflexão crítica sobre suas práticas diárias e como essa reflexão é facilitada por ferramentas educacionais, como diários de campo, fóruns de discussão e encontros síncronos. A prática reflexiva oportuniza que os profissionais identifiquem áreas de melhoria e adaptem suas abordagens para atender melhor às necessidades das pessoas com condições crônicas^(11)^.

A atividade de educação permanente em saúde, por meio da problematização de situações cotidianas frente às condições crônicas e metodologias de grupo, motiva os profissionais de saúde. Diante do problema, eles se detêm, examinam, refletem, relacionam a sua história e passam a ressignificar suas descobertas. As atividades de educação permanente alinhadas à prática dos serviços de saúde, tendo como base metodologias em grupo, possibilitam a liberdade e a autonomia na realização de escolhas e na tomada de decisões. É necessário inovar para abordagens educacionais ativas e problematizadoras^(11)^.

Os profissionais egressos do Curso de especialização relataram diversas dificuldades e limitações na gestão e assistência às condições crônicas, como a falta de recursos humanos e a necessidade de adaptação às condições locais. A fala “Não tem nutricionista, sabe? Tem uma nutricionista por região, essa nutricionista tem que cuidar de quatro bairros (N2)” exemplifica a sobrecarga de trabalho e a insuficiência de recursos. Esses desafios impactam diretamente a qualidade do cuidado prestado e a capacidade de implementar estratégias preventivas^(12)^.

A prevenção das condições crônicas é reconhecida como uma estratégia essencial, mas os profissionais apontam que o foco atual está predominantemente no cuidado curativista. A fala “Hoje em dia a gente acaba focando no paciente quando a doença já está instalada e eu acredito que a prevenção seria o melhor caminho (N2)” destaca essa preocupação. Além disso, a revisão contínua de conhecimentos é vista como uma estratégia para melhorar a prática profissional; conforme ilustrado no recorte, as leituras ajudaram a gente a conseguir escrever, responder, tudo isso (E17).

Existe um predomínio no modelo de atenção prescritivo, focado na doença e com pouca atuação das equipes na atenção integral da saúde e no empoderamento dos usuários. A melhoria da saúde das pessoas com condições crônicas demanda a transformação de sistemas essencialmente reativos (que respondem principalmente quando uma pessoa está doente) em sistemas pró-ativos e concentrados em manter a pessoa o mais saudável possível^(13,14)^.

As condições crônicas exigem um cuidado contínuo e uma educação permanente tanto para a equipe de saúde quanto para os pacientes. Esse processo é fundamental para esclarecer dúvidas sobre o tratamento em andamento e para abordar questões que possam surgir durante a adesão ao tratamento. A troca de informações entre o paciente e a unidade de saúde é essencial para construir uma relação de confiança e eficácia. A continuidade desse cuidado, orientada por diretrizes e protocolos estabelecidos pela equipe de saúde, resulta em diagnósticos e tratamentos mais precisos. Além disso, garante um direcionamento adequado durante toda a assistência, proporcionando um atendimento personalizado e eficaz para cada indivíduo^(14–16)^.

Gerenciar a aprendizagem e o conhecimento nas instituições de saúde representa um desafio significativo para os profissionais da área. A elaboração de estratégias eficazes para a criação, manutenção e compartilhamento de informações e conhecimentos é essencial. Essas estratégias permitem que os profissionais de saúde acessem rapidamente as alternativas necessárias para tomar decisões informadas e eficazes^(17)^.

O feedback reflexivo, obtido por meio da interação nos fóruns e encontros presenciais, contribui para a melhoria das práticas profissionais, como se pode observar nas falas: *A troca também com outras pessoas, outras experiências ali para participar dos debates, dos fóruns, foi bem interessante* (E15). *Esses encontros, eu sempre acho muito rico para aprender e trocar* (E11). Isso destaca a importância da troca de experiências e ideias entre profissionais de diferentes municípios e contextos. Esses momentos de troca e feedback contribuem para uma análise mais rica e detalhada das experiências, ajudando os profissionais a identificarem áreas que podem ser aprimoradas e a implementar mudanças efetivas. Além disso, uma revisão dos resultados do aprendizado e a avaliação de abordagens alternativas para resolver problemas auxiliam os especialistas a aplicarem o conhecimento adquirido para desenvolver estratégias eficazes no manejo das pessoas com condições crônicas, no território^(17)^.

### Observação de Mudanças

A capacidade de observar e refletir sobre mudanças nas práticas evidenciou o papel do curso na adaptação profissional a cenários dinâmicos de saúde. A reflexão crítica sobre os resultados alcançados demonstra o impacto positivo do aprendizado reflexivo na consolidação de práticas baseadas em evidências. Estudos recentes afirmam que a educação baseada na reflexão crítica melhora a capacidade dos profissionais de responder a situações complexas no ambiente de trabalho^(3)^.

A reflexão pessoal, que trouxe à tona angústias relacionadas às limitações enfrentadas na prática, também é um tema comum na literatura, onde a falta de recursos e a sobrecarga de trabalho são frequentemente citadas como desafios significativos^(18,19)^. A análise de casos e a discussão de desafios no curso foram apontadas como ferramentas valiosas para contextualizar o conhecimento teórico na prática diária.

A troca de experiências nos fóruns de discussão ajudou a identificar problemas comuns e soluções inovadoras, evidenciando a importância da observação crítica e da reflexão na melhoria contínua das práticas de saúde. Essa prática de “reflexão-na-ação” é essencial para a melhoria contínua das práticas de saúde, pois permite que os profissionais adaptem suas abordagens com base em experiências e feedbacks^(20)^.

A capacidade dos profissionais de analisar suas experiências práticas e identificar áreas de melhoria foi um aspecto destacado por uma subcategoria. A reflexão sobre experiências passadas é fundamental para o aprendizado contínuo e a adaptação de práticas^(21,22)^. A literatura também sugere que a capacidade de observar e refletir sobre mudanças é importante para a implementação de melhorias nas práticas de saúde^(23)^.

### Feedback e Melhorias

O feedback foi destacado como elemento essencial para a autoavaliação e ajustes na prática. A interação em fóruns e encontros presenciais fomentou discussões enriquecedoras, permitindo que os profissionais identificassem lacunas e implementassem estratégias inovadoras. Esse achado está alinhado com a literatura, que posiciona o feedback como ferramenta poderosa para a melhoria contínua.

O feedback recebido dos tutores e colegas foi considerado valioso para ajustes na prática profissional. A literatura destaca que o feedback construtivo é um componente crítico do aprendizado reflexivo, pois permite aos profissionais identificarem áreas de melhoria e desenvolver estratégias para abordar essas áreas^(24)^. O feedback é uma das ferramentas mais poderosas para a melhoria contínua, pois oferece ideias valiosas sobre as práticas atuais e possíveis melhorias^(25)^.

O planejamento de melhorias e a autoavaliação emergiram como componentes essenciais para o desenvolvimento profissional contínuo. Estudos recentes afirmam que a autoavaliação ajuda os profissionais a reconhecerem suas próprias competências e limitações, promovendo um ciclo de melhoria contínua^(26)^. A autoavaliação é fundamental para o desenvolvimento de uma prática reflexiva, onde os profissionais estão constantemente avaliando e ajustando suas abordagens para melhor atender às necessidades dos pacientes^(21)^.

A partilha de experiências nos fóruns e encontros presenciais permitiu uma reflexão coletiva, essencial para ampliar o entendimento sobre as práticas de cuidado. Este achado é corroborado por estudos que mostram que a colaboração entre profissionais de diferentes áreas de especialização enriquece a prática e promove uma abordagem mais holística ao cuidado. A aprendizagem situada em comunidades de prática oferece uma rica troca de conhecimentos e experiências que é fundamental para a melhoria contínua^(27)^.

### Revisão Crítica e Planejamento

A subcategoria destaca a importância de revisar estratégias e planejar ações com base em desafios práticos. Ferramentas colaborativas, como fóruns e discussões presenciais, facilitaram o aprofundamento do conhecimento e a inovação nas práticas de cuidado.

Na subcategoria “Revisão Crítica e Planejamento”, os egressos enfatizaram a importância da revisão de estratégias e da reflexão sobre dificuldades enfrentadas na prática. A literatura sugere que a revisão crítica de estratégias permite que os profissionais ajustem suas abordagens com base em evidências e melhores práticas^(28)^. Esta prática é essencial para garantir que os cuidados prestados sejam sempre os mais eficazes e baseados nas melhores evidências disponíveis.

A revisão de materiais e a discussão de técnicas foram métodos eficazes para aprofundar o conhecimento e aprimorar as práticas profissionais. O aprendizado contínuo e a revisão de materiais didáticos são essenciais para manter-se atualizado com as melhores práticas e evidências mais recentes^(21)^. A revisão crítica e a discussão de técnicas são fundamentais para a inovação e a melhoria das práticas de saúde^(23)^.

A revisão de processos foi identificada como uma etapa importante para consolidar o aprendizado e garantir a melhoria contínua das práticas de saúde. Este achado é suportado por estudos que mostram que a revisão regular de processos permite que os profissionais identifiquem ineficiências e implementem melhorias^(1)^. Além disso, a revisão sistemática dos processos é essencial para a implementação de práticas baseadas em evidências e para a adaptação às mudanças nas necessidades dos pacientes^(29)^.

### Limitações e Perspectivas Futuras

Este estudo apresenta limitações relacionadas à generalização dos resultados, devido ao foco exclusivo em egressos de um curso específico. Pesquisas futuras podem explorar longitudinalmente o impacto da formação reflexiva e comparar diferentes abordagens pedagógicas, aprofundando a compreensão das competências reflexivas em saúde.

Embora este estudo tenha proporcionado resultados valiosos sobre o desenvolvimento de competências reflexivas e críticas por profissionais da saúde, é importante reconhecer algumas limitações. Primeiro, a amostra foi composta exclusivamente por egressos de um curso específico de especialização em doenças crônicas não-transmissíveis, o que pode limitar a generalização dos resultados para outras áreas de especialização ou contextos educacionais. Segundo, a natureza qualitativa do estudo, baseada em entrevistas semiestruturadas, pode estar sujeita a vieses de autorrelato e interpretação dos dados, ainda que as mesmas tenham sido validadas pelos próprios participantes da pesquisa.

Além disso, este estudo focou predominantemente na dimensão da Observação Reflexiva do Ciclo de Aprendizagem Experiencial de Kolb, deixando de explorar outras dimensões do ciclo, como a experiência concreta, a experimentação ativa e a conceituação abstrata, que também são importantes para o desenvolvimento completo de competências profissionais.

E, por fim, pesquisas futuras poderiam explorar longitudinalmente o impacto das competências desenvolvidas ao longo do tempo, avaliando como a formação reflexiva influencia a prática profissional e a qualidade do atendimento em longo prazo. Estudos comparativos entre diferentes programas de formação e métodos pedagógicos também seriam valiosos para identificar as abordagens mais eficazes para o desenvolvimento de competências críticas e reflexivas entre profissionais de saúde.

## CONCLUSÃO

Este estudo sublinha a importância da problematização, reflexão crítica e da OR na formação de profissionais de saúde, particularmente no contexto do manejo de CCNT, meio de metodologias que incentivam a análise profunda das práticas diárias, como diários de campo, fóruns de discussão e encontros síncronos. Foi possível identificar melhorias significativas nas abordagens de cuidado. Estas ferramentas educacionais não apenas facilitam o aprendizado colaborativo, mas também promovem uma cultura de autoavaliação e aprimoramento contínuo entre os profissionais.

A educação permanente em saúde, por meio da problematização de situações cotidianas e metodologias de grupo, motiva os profissionais de saúde a refletirem sobre suas práticas e a ressignificarem suas descobertas. A troca de experiências e ideias entre profissionais de diferentes contextos contribui para uma análise mais rica e detalhada das experiências, promovendo a implementação de mudanças efetivas.

A integração da TAE de Kolb no currículo de especialização demonstrou ser um modelo eficaz para desenvolver competências críticas e reflexivas. Este enfoque abre portas para que os profissionais de saúde adaptem suas práticas às realidades específicas de seus territórios, melhorando a qualidade do atendimento e respondendo de forma mais precisa às necessidades individuais e coletivas.

No entanto, o estudo também revela desafios persistentes, como a sobrecarga de trabalho e a escassez de recursos, que impactam a implementação de estratégias preventivas e a atenção integral à saúde. Esses obstáculos evidenciam a necessidade de políticas públicas mais robustas e investimentos contínuos na formação e suporte aos profissionais de saúde.

# Translation: Competencia de los profesionales de la salud para problematizar y reflexionar sobre situaciones de personas con condiciones crónicas*

**DOI:** 10.1590/1980-220X-REEUSP-2024-0314es

**Authors:** Thaise Honorato de Souza, Saulo Fabio Ramos, Janaína da Silva Flôr, Joice Cristina Guesser, Natália Gonçalves, Vânia Marli Schubert Backes, Roberta Waterkemper, Monica Motta Lino

**Affiliations:** 1 Universidade Federal de Santa Catarina, Programa de Pós-graduação em Enfermagem, Florianópolis, SC, Brasil.

**Keywords:** Competencia Profesional, Atención Integral de Salud, Enfermedades no Transmisibles, Insuficiencia Renal Crónica, Educación Continua

## Abstract

**Objetivo:**

Evaluar el impacto del Curso de Especialización en Atención a Personas con Enfermedades Crónicas No Transmisibles en el quehacer de los profesionales de la salud, centrado en la competencia para problematizar y reflexionar sobre situaciones de personas con padecimientos crónicos.

**Método:**

Evaluación ex-post facto basada en el Ciclo de Aprendizaje Experiencial, con énfasis en la observación reflexiva. Realizada entre mayo de 2023 y junio de 2024, la investigación siguió las directrices del COREQ e incluyó entrevistas semiestructuradas a 41 graduados, previa aprobación ética. Se identificaron 40 códigos, 4 subcategorías y un eje central. Financiación: CNPq y FAPESC.

**Resultados:**

La observación reflexiva, según la Teoría del Aprendizaje Experiencial, fue fundamental para el desarrollo de habilidades de problematización y reflexión sobre situaciones y contextos vividos por personas con enfermedades crónicas.

**Conclusión:**

El estudio destaca la importancia de la observación reflexiva en el desarrollo de competencias para el manejo de condiciones crónicas y la integración de la Teoría del Aprendizaje Experiencial en la formación de profesionales de salud para mejorar la atención.

## INTRODUCCIÓN

David Kolb, en su Teoría del Aprendizaje Experiencial (TAE), enfatiza la importancia de las experiencias prácticas en la construcción de las habilidades y conocimientos necesarios para resolver problemas, destacando que «el conocimiento se construye a partir de la transformación de la experiencia»^(1)^.

La Observación Reflexiva (OR), de la que se habla en este artículo, es una de las partes del Ciclo de Kolb. Se refiere a la problematización y expresa la capacidad de examinar, investigar y medir o reflexionar críticamente sobre situaciones, hechos o contextos vividos en el territorio por personas con enfermedades crónicas. Se centra en la reflexión y la observación de las experiencias vividas. Implica observar atentamente los problemas y tratar de comprender cómo se producen los acontecimientos. El desarrollo de esta competencia permite a los profesionales revisar casos clínicos, mantener discusiones de grupo o escribir diarios reflexivos^(1)^. La competencia es la capacidad de un profesional para realizar una tarea específica, respaldada por una formación y una educación adecuadas^(2)^.

Estudios recientes destacan la importancia de desarrollar la competencia para problematizar y reflexionar críticamente sobre las prácticas sanitarias. La capacidad de reflexión crítica no sólo mejora la toma de decisiones, sino que también aumenta la calidad de la atención al paciente^(3)^. Además, promover la reflexión crítica y la problematización en el contexto de la educación en salud es fundamental para formar profesionales más independientes y eficientes. Estas competencias son relevantes para afrontar los retos de un entorno sanitario en constante cambio y para promover intervenciones basadas en la evidencia.

Una práctica sanitaria eficaz requiere la integración de conocimientos, habilidades y actitudes adecuados a las realidades y necesidades sanitarias de la población. Una práctica profesional eficiente debe basarse en acciones que impliquen autonomía, ética y capacidad para diagnosticar y resolver problemas en el ámbito de la atención sanitaria y la gestión de cuidados^(4)^. La calidad de los servicios sanitarios depende de cómo los profesionales combinan sus valores, conocimientos, habilidades y comportamientos para responder a las necesidades de la comunidad^(5)^.

Este estudio forma parte de la tesis doctoral en enfermería, vinculada al Macroproyecto «Evaluación del impacto tras la cualificación profesional de trabajadores, gestores y especialistas en enfermedades crónicas no transmisibles», y tiene como objetivo evaluar el impacto en el proceso de trabajo de los profesionales sanitarios egresados del «Curso de Especialización en Atención a Personas con Enfermedades Crónicas No Transmisibles (CCNT)», centrándose en la competencia para problematizar y reflexionar sobre las situaciones y contextos que viven las personas con enfermedades crónicas en el territorio. La investigación se basa en el Ciclo de Aprendizaje Experiencial de Kolb, con énfasis en la dimensión OR.

## MÉTODO

### Diseño del Estudio

Una evaluación de impacto cualitativa ex-post-facto, a la luz de la TAE, centrada en la OR de los profesionales sanitarios que se han graduado en un Curso de Especialización. El estudio utilizó el instrumento *Consolidated criteria for reporting qualitative research* (COREQ)^(6)^ para comprobar todas las etapas del método.

### Lugar del Estudio

El estudio fue realizado en seis municipios del estado de Santa Catarina: Blumenau, Chapecó, Criciúma, Florianópolis, Joinville y Lages.

## POBLACIÓN

El «Curso de Especialización en Atención a Personas con Enfermedades Crónicas No Transmisibles», promovido por la UFSC entre 2023 y 2024, capacitó a profesionales de salud del SUS en Santa Catarina, utilizando un abordaje híbrido y la Teoría del Aprendizaje Experiencial de Kolb. Con 360 horas, divididas entre el Eje Integrador, que abordaba las políticas públicas y la promoción de la salud, y el Eje Especializado, centrado en la gestión de enfermedades crónicas como la diabetes y la hipertensión, el curso fomentaba la aplicación práctica del aprendizaje a través de proyectos de intervención.

La encuesta incluyó a 337 profesionales sanitarios graduados en el Curso de Especialización. La muestra fue intencionalmente estratificada y distribuida por polos y diferentes profesiones de la salud, incluyendo 41 entrevistas a egresados del curso (12%), lo que saturó teóricamente el tema.

### Recolección de Datos

La recolección de datos se realizó de junio a septiembre de 2023 e incluyó las siguientes etapas: (i) invitación y aceptación: enviada vía WhatsApp® a los profesionales egresados del Curso de Especialización; (ii) programación y realización de las entrevistas: con programación previa y realizadas virtualmente; (iii) realización de la entrevista por el investigador: el día programado se envió el link de acceso, seguido de la lectura de los objetivos de la investigación, solicitud de consentimiento para la realización de la entrevista y presentación del guión semiestructurado abordando temas como experiencias durante el curso, cambios en las prácticas profesionales y aplicación de los conocimientos en la práctica clínica. Las entrevistas tuvieron una duración media de 40 minutos y fueron grabadas mediante Google Meet® y posteriormente transcritas.

### Análisis y Tratamiento de los Datos

Los datos fueron sometidos a análisis temático de contenido^(7)^, que comprende tres fases, a la luz de la TAE de Kolb: 1) preanálisis, en el que se organizó el material, seguido de 2) exploración, en el que se agregó la información en categorías temáticas y, por último, 3) tratamiento de los resultados brutos y su interpretación, para luego proponer inferencias, todo lo cual fue realizado manualmente por el investigador. Las 206 páginas de material se analizaron entre junio de 2023 y junio de 2024. Surgieron 40 códigos, que dieron como resultado cuatro subcategorías y un eje central de análisis: la dimensión OR del Ciclo de Aprendizaje Experiencial. En el Curso, OR se refiere a la problematización y expresa la capacidad de analizar, investigar, medir, problematizar y reflexionar críticamente sobre situaciones, hechos o contextos vividos en el territorio por personas con condiciones crónicas.

### Aspectos Éticos

Artículo aprobado por el Comité de Ética en Investigación de la UFSC, según el dictamen N6.078.402. La investigación cumple con la Resolución 466 del 12 de diciembre de 2012. Para preservar la identidad de los participantes, se han adoptado seudónimos de acuerdo con la profesión y el número secuencial, como Fi1 (Fisioterapeuta 1).

## RESULTADOS

Un total de 41 (12%) profesionales de salud fueron entrevistados en seis centros del estado de Santa Catarina/SC (Tabla 1).

**Tabla 1:** Caracterización de los participantes del estudio según polo, profesión y tema del Trabajo de Conclusión del Curso (TCC) – Florianópolis, SC, Brasil, 2024 (n = 41).

Polo	n	Profesión	Tema del TCC*.
Blumenau	5	04 Enfermeras 01 Farmacéutico	Asistencia farmacéutica, cáncer de cuello de útero, DM, HAS
Chapecó	5	01 trabajador social 01 Dentista 02 Enfermeras 01 Farmacéutico	DM, HAS, obesidad
Criciúma	11	01 trabajador social 05 Enfermeras 02 Farmacéuticos 01 Fisioterapeuta 01 Nutricionista 01 Psicólogo	DM, HAS, obesidad, salud mental, tabaquismo
Florianópolis	6	05 Enfermeras 01 Nutricionista	DM, HAS, obesidad, salud mental, tabaquismo
Joinville	7	04 Enfermeras 01 Fisioterapeuta 01 Médico 01 Nutricionista	DM, HAS
Lages	7	01 Educador físico 05 Enfermeras 01 Fisioterapeuta	Actividad física, depresión, HAS, obesidad, tabaquismo

DM: diabetes Mellitus HAS: Hipertensión Arterial Sistémica

El análisis de las entrevistas transcritas, basado en el Ciclo de Aprendizaje Experiencial de Kolb, dio lugar a la identificación de 152 códigos, que se organizaron en 16 subcategorías y 4 categorías, según sus similitudes.

La categoría Experiencia Concreta (EC) destacó elementos relacionados con la experiencia práctica de los participantes, incluyendo la participación y el impacto inicial del curso, el uso de materiales y métodos de enseñanza, la interacción y el apoyo entre profesionales, así como los retos afrontados al inicio del proceso de formación. En la categoría Observación Reflexiva (OR), se destacaron las reflexiones críticas sobre la práctica profesional, los intercambios de feedback y las sugerencias de mejora, así como la identificación de los cambios observados en los contextos locales y la planificación estratégica basada en estas observaciones.

La categoría Conceptualización Abstracta (CA) abordó la sistematización del aprendizaje, destacando el desarrollo teórico de los participantes, la aplicación de los conceptos aprendidos en contextos prácticos, la construcción y comprobación de hipótesis y la integración de los nuevos conocimientos con las prácticas existentes. Finalmente, la categoría Experimentación Activa (EA) reflejó la aplicación práctica de los conocimientos adquiridos, abarcando la implementación y adaptación de estrategias, el desarrollo y prueba de intervenciones, la búsqueda de mejoras continuas y soluciones prácticas, así como el uso de los conocimientos teóricos en acciones concretas.

En este artículo, destacaremos la categoría «Observación reflexiva», que surgió de los datos en consonancia con el marco TAE de Kolb.

La categoría «Observación Reflexiva» emergió como una dimensión central en el desarrollo de las competencias de los profesionales de la salud egresados del «Curso de Especialización en Atención a Personas con Enfermedades Crónicas No Transmisibles». Basada en el Ciclo de Aprendizaje Experiencial, esta competencia, adquirida durante el Curso de Especialización y destacada en esta categoría, permitió a los profesionales reflexionar críticamente sobre las situaciones y contextos vividos por las personas con enfermedades crónicas. Al problematizar sus prácticas, los profesionales pudieron desarrollar nuevos enfoques asistenciales y mejorar la calidad de la atención.

Dentro de esta categoría, surgieron 40 códigos y cuatro subcategorías: Reflexión sobre la Práctica, Retroalimentación y Mejora, Observación del Cambio y Revisión Crítica y Planificación (Tabla 2).

**Tabla 2:** Categoría, subcategorías y códigos de la Observación Reflexiva – Florianópolis, SC, Brasil, 2024.

Subcategoría	Códigos
Reflexión sobre la práctica	Reflexión sobre la práctica, Evaluación crítica del curso, Reflexión personal, Análisis de casos, Discusión de retos, Revisión de conocimientos previos, Comparación con experiencias anteriores, Identificación de lagunas, Revisión de objetivos, Reflexión sobre errores, Reflexión sobre feedback recibido, Reflexión sobre dificultades, Reflexión sobre aprendizaje, Análisis de intervenciones.
Comentarios y mejoras	Retroalimentación reflexiva, Reflexión sobre la retroalimentación recibida, Ajustes a la práctica profesional, Discusión de la experiencia, Planificación de mejoras, Autoevaluación, Revisión de estrategias, Revisión de procesos, Revisión de resultados, Análisis de la retroalimentación, Revisión de materiales, Revisión de literatura, Discusión de mejoras, Planificación de nuevos enfoques.
Observar los cambios	Observación del cambio, Identificación del éxito, Revisión de los resultados, Discusión de enfoques alternativos, Reflexión sobre los resultados, Observación de las prácticas de los colegas, Observación de prácticas eficaces, Observación de tendencias, Identificación de buenas prácticas, Reflexión sobre el impacto.
Revisión crítica y planificación	Revisión de estrategias, Reflexión sobre dificultades, Revisión de materiales, Discusión de técnicas, Revisión de procesos, Discusión de innovaciones, Discusión de técnicas, Discusión de casos, Discusión de experiencias, Discusión de retos.

### Reflexión Sobre la Práctica

Dentro de la subcategoría «Reflexión sobre la Práctica», los graduados informaron que el uso de diarios fue una herramienta importante para llevar la teoría a la práctica en el municipio, permitiéndoles reflexionar profundamente sobre sus acciones en el contexto de CCNT. Por ejemplo, un profesional comentó: *En relación a los diarios, es algo que nos ayuda a llevar la realidad del municipio, nos hace reflexionar sobre lo que ya practicamos y lo que se puede mejorar* (Fi3). Esta reflexión continua permitió a los profesionales evaluar críticamente sus prácticas e identificar áreas de mejora.

La evaluación crítica del curso también fue un aspecto significativo en esta subcategoría. Los participantes reconocieron la utilidad del curso para mejorar la gestión de las personas con ENT, como se mencionó: *El curso ayudó, fue muy útil en esa parte también* (Fa3). Las reflexiones personales sacaron a relucir ansiedades relacionadas con las limitaciones a las que se enfrentan en la práctica profesional, como la falta de recursos o de personal especializado. Una nutricionista destacó: *No hay nutricionistas, ¿sabes? Hay un nutricionista por región, y ese nutricionista tiene que atender cuatro barrios* (N2).

El análisis de casos y la discusión de desafíos durante el curso se consideraron herramientas valiosas para contextualizar los conocimientos teóricos en la práctica diaria. *El intercambio de experiencias en los foros de discusión ayudó a identificar problemas comunes y soluciones innovadoras, destacando la importancia de la observación crítica y la reflexión en la mejora continua de las prácticas sanitarias. Hubo interacción entre compañeros* (...) *intercambio de experiencias* (Fi1).

El alumno valora y reflexiona sobre la importancia de repasar los conocimientos aprendidos para mejorar su práctica con personas con ENTs: *Los conocimientos para mí trajeron más seguridad en relación a mi actuación profesional y personal, me sentí más seguro a la hora de orientar, acompañar. Aporta más seguridad* (...) *apoyo* (E16).

### Feedback y Mejoras

La subcategoría «Retroalimentación y mejoras» destacó la importancia de la interacción en los foros y el intercambio de ideas como forma de enriquecer la reflexión individual y colectiva. Un participante *comentó El intercambio también con otras personas, otras experiencias allí para participar en los debates, los foros, fue muy interesante* (E15PCr). Esta interacción permitió comparar prácticas e incorporar nuevas ideas, promoviendo un aprendizaje continuo y colaborativo.

La retroalimentación recibida de los tutores y colegas fue considerada valiosa para la autoevaluación y ajustes de la práctica profesional. Los tutores y los encuentros presenciales fueron mencionados como momentos importantes para la reflexión crítica: *Estos encuentros, siempre pienso, son muy ricos* (...) *para aprender e intercambiar* (E11).

Debatir las experiencias con otros profesionales permitió analizar las prácticas en detalle e identificar las áreas que debían mejorarse, como puede verse en este informe: *El foro creo que es muy importante porque como estamos tan centrados en nuestra región, en nuestra ciudad, no observamos a los demás. Y a veces lo que funciona en una ciudad puede funcionar para mí* (E19).

La planificación de mejoras se vio facilitada por el uso de diarios y la reflexión continua sobre las prácticas actuales: *En cuanto a los diarios, es algo que nos ayuda a poner en juego la realidad del municipio, nos hace reflexionar sobre lo que ya practicamos y lo que se puede mejorar* (Fi3).

La autoevaluación surgió como un componente esencial para el desarrollo profesional, que permite a los graduados identificar sus puntos fuertes y débiles y tratar constantemente de mejorar sus competencias. *Así que esta especialización fue muy gratificante, porque contribuyó a mi aprendizaje y para que pudiera reforzar ese aprendizaje* (E25).

### Observación de los Cambios

La subcategoría «Observación de los cambios» puso de relieve la capacidad de los profesionales para analizar sus experiencias prácticas, identificar las áreas de mejora y consolidar su aprendizaje. *Aprendí mucho durante este curso de un año, que también me ayudó a gestionar mejor a los pacientes, a explicarles ciertas cosas. Hablar con los pacientes y explicarles las cosas me ayudó mucho* (Fa3). Compartir experiencias en foros y reuniones presenciales proporcionó un espacio de reflexión colectiva, esencial para ampliar la comprensión de las prácticas asistenciales: *La reunión presencial también fue muy rica por el intercambio de experiencias, al ser un curso de postgrado con mucha gente de otras especialidades* (E17).

Revisar los resultados y discutir enfoques alternativos permitió a los profesionales evaluar diferentes perspectivas y adaptar sus prácticas según fuera necesario. Reflexionar sobre los resultados obtenidos fue importante para validar el proceso de aprendizaje e implementar cambios significativos en las prácticas asistenciales: *Me ayudó mucho a entender algunas cosas, el manejo del paciente, el trato con los diferentes tipos de pacientes que tenemos* (Fa3).

### Revisión Crítica y Planificación

En la subcategoría «Revisión crítica y planificación», los graduados destacaron la importancia de revisar las estrategias y reflexionar sobre las dificultades encontradas en la práctica. El quirófano permitió a los profesionales analizar sus experiencias, identificar áreas de mejora y consolidar el aprendizaje. Un participante destacó: *Hoy en día acabamos centrándonos en el paciente cuando la enfermedad ya se ha instalado y creo que la prevención sería el mejor camino a seguir* (N2).

La revisión de materiales y la discusión de técnicas se mencionaron como métodos eficaces para profundizar en los conocimientos y mejorar las prácticas profesionales. Los foros de debate, las clases sincrónicas y las reuniones presenciales animaron a los profesionales a compartir ideas y soluciones, promoviendo un entorno de aprendizaje colaborativo: *El intercambio con otras personas, otras experiencias allí para participar en los debates, los foros, fue muy interesante* (E15).

La revisión de los procesos se identificó como una etapa importante para consolidar el aprendizaje y garantizar la mejora continua de las prácticas sanitarias: *Todo lo que aprendemos de alguna manera cambia nuestra visión, nuestra perspectiva de las cosas del día a día, sobre todo en sanidad* (E15). Por lo tanto, la observación reflexiva no sólo promovió el desarrollo de competencias críticas, sino que también facilitó la adaptación y la innovación en las prácticas sanitarias.

## DISCUSIÓN

El estudio muestra que la formación profesional en salud en el curso objeto de estudio, basada en la TAE de Kolb, potencia el desarrollo de competencias críticas reflexivas a través de estrategias como los diarios de campo, los foros de discusión, el análisis de casos y el feedback colaborativo, permitiendo a los profesionales conectar teoría y práctica, identificar sus limitaciones y desarrollar prácticas más adaptativas e innovadoras, desarrollar prácticas más adaptativas e innovadoras, y promover un enfoque más holístico y contextualizado de la atención a las enfermedades crónicas, superando el modelo tradicional prescriptivo y curativo mediante el fomento de una práctica profesional basada en la reflexión continua, el intercambio de experiencias y el replanteamiento constante de los conocimientos.

### Reflexión Sobre la Práctica

Los profesionales sanitarios señalaron que herramientas como los diarios y la discusión de casos les permitían conectar teoría y práctica, promoviendo una revisión crítica continua. Este resultado refuerza la importancia de la reflexión para la práctica transformadora, tal como se describe en la TAE de Kolb. Estudios similares corroboran la eficacia de las estrategias reflexivas en la mejora de la atención clínica, que permite a los profesionales cuestionar sus propios supuestos y prejuicios, lo que conduce a un aprendizaje transformador y al desarrollo de prácticas más eficaces ^(8)^.

Kolb subraya la importancia de considerar múltiples perspectivas durante la reflexión, lo que significa que los expertos deben contemplar la experiencia desde diferentes ángulos y considerar diversas interpretaciones y significados^(1)^. Este enfoque permite a los profesionales desarrollar una comprensión más profunda de las mejores prácticas asistenciales, lo que resulta esencial para la gestión eficaz de las enfermedades crónicas.

La interacción en los foros y el intercambio de ideas fueron importantes para el aprendizaje continuo, según informaron los graduados. Este tipo de aprendizaje colaborativo está reconocido en la literatura como beneficioso para el desarrollo profesional. Las comunidades de práctica, como los foros de debate, proporcionan un espacio para que los profesionales compartan experiencias, resuelvan problemas comunes y desarrollen nuevas competencias^(9,10)^.

La OR, tal y como se describe en la TAE de Kolb, es fundamental para desarrollar competencias de problematización y reflexión sobre situaciones y contextos vividos por personas con enfermedades crónicas. Los profesionales egresados del curso de especialización en ENT destacaron la importancia de la reflexión crítica sobre sus prácticas cotidianas y cómo esta reflexión es facilitada por herramientas educativas como los diarios de campo, los foros de discusión y las reuniones sincrónicas. La práctica reflexiva permite a los profesionales identificar áreas de mejora y adaptar sus enfoques para satisfacer mejor las necesidades de las personas con enfermedades crónicas^(11)^.

La formación sanitaria continuada, mediante la problematización de situaciones cotidianas relacionadas con las enfermedades crónicas y las metodologías de grupo, motiva a los profesionales sanitarios. Ante el problema, se detienen, examinan, reflexionan, relatan su historia y empiezan a resignificar sus conclusiones. Las actividades de formación continuada alineadas con la práctica de los servicios sanitarios, basadas en metodologías de grupo, permiten libertad y autonomía en la toma de decisiones y elecciones. Es necesario innovar hacia abordajes educativos activos y problematizadores^(11)^.

Los profesionales egresados del curso de especialización relataron diversas dificultades y limitaciones en el manejo y cuidado de las condiciones crónicas, como la falta de recursos humanos y la necesidad de adaptación a las condiciones locales. «No hay nutricionista. Hay un nutricionista por región, y ese nutricionista tiene que atender cuatro barrios (N2)» ejemplifica la sobrecarga de trabajo y la insuficiencia de recursos. Estas dificultades repercuten directamente en la calidad de la atención prestada y en la capacidad de aplicar estrategias preventivas^(12)^.

La prevención de las enfermedades crónicas se reconoce como una estrategia esencial, pero los profesionales señalan que la atención actual se centra predominantemente en los cuidados curativos. La afirmación «Hoy en día acabamos centrándonos en el paciente cuando la enfermedad ya se ha instalado y creo que la prevención sería el mejor camino a seguir (N2)» pone de manifiesto esta preocupación. Además, la revisión continua de conocimientos es vista como una estrategia para mejorar la práctica profesional; como se ilustra en el recorte, las lecturas nos ayudaron a saber escribir, contestar, todo eso (E17).

Predomina el modelo de atención prescriptivo, centrado en la enfermedad y con escasa actuación de los equipos en la atención integral de la salud y en el empoderamiento de los usuarios. Mejorar la salud de las personas con enfermedades crónicas requiere transformar sistemas esencialmente reactivos (que responden principalmente cuando una persona está enferma) en sistemas proactivos centrados en mantener a la persona lo más sana posible^(13–14)^.

Las afecciones crónicas requieren una atención continua y una educación permanente tanto para el equipo sanitario como para los pacientes. Este proceso es fundamental para aclarar dudas sobre el tratamiento en curso y para abordar los problemas que puedan surgir durante la adherencia al tratamiento. El intercambio de información entre el paciente y la unidad sanitaria es esencial para construir una relación de confianza y eficacia. La continuidad de esta atención, guiada por pautas y protocolos establecidos por el equipo sanitario, redunda en diagnósticos y tratamientos más precisos. Además, garantiza una orientación adecuada a lo largo de la atención, proporcionando cuidados personalizados y eficaces para cada individuo^(14–16)^.

La gestión del aprendizaje y el conocimiento en las instituciones sanitarias representa un reto importante para los profesionales del sector. Es esencial desarrollar estrategias eficaces para crear, mantener y compartir información y conocimientos. Estas estrategias permiten a los profesionales sanitarios acceder rápidamente a las alternativas que necesitan para tomar decisiones informadas y eficaces^(17)^.

La retroalimentación reflexiva, obtenida a través de la interacción en foros y reuniones presenciales, contribuye a mejorar las prácticas profesionales, como puede verse en los comentarios: *El intercambio también con otras personas, otras experiencias allí para participar en los debates, los foros, fue muy interesante* (E15). *Siempre encuentro estas reuniones muy útiles para aprender e intercambiar* (E11). Esto pone de manifiesto la importancia del intercambio de experiencias e ideas entre profesionales de diferentes municipios y contextos. Estos momentos de intercambio y retroalimentación contribuyen a un análisis más rico y detallado de las experiencias, ayudando a los profesionales a identificar áreas susceptibles de mejora y a implementar cambios efectivos. Además, la revisión de los resultados del aprendizaje y la evaluación de enfoques alternativos para resolver problemas ayudan a los especialistas a aplicar los conocimientos adquiridos para desarrollar estrategias eficaces de gestión de las personas con enfermedades crónicas en el territorio^(17)^.

### Observación del Cambio

La capacidad de observar y reflexionar sobre los cambios en la práctica puso de manifiesto el papel del curso en la adaptación profesional a escenarios sanitarios dinámicos. La reflexión crítica sobre los resultados obtenidos demuestra el impacto positivo del aprendizaje reflexivo en la consolidación de prácticas basadas en la evidencia. Estudios recientes afirman que la educación basada en la reflexión crítica mejora la capacidad de los profesionales para responder a situaciones complejas en el lugar de trabajo^(3)^.

La reflexión personal, que sacó a la luz ansiedades relacionadas con las limitaciones a las que se enfrentan en la práctica, es también un tema común en la literatura, donde la falta de recursos y la sobrecarga de trabajo se citan a menudo como retos significativos^(18,19)^. El análisis de casos y la discusión de retos durante el curso se consideraron herramientas valiosas para contextualizar los conocimientos teóricos en la práctica diaria.

El intercambio de experiencias en los foros de debate ayudó a identificar problemas comunes y soluciones innovadoras, destacando la importancia de la observación crítica y la reflexión en la mejora continua de las prácticas sanitarias. Esta práctica de «reflexión en la acción» es esencial para la mejora continua de las prácticas sanitarias, ya que permite a los profesionales adaptar sus enfoques en función de las experiencias y la retroalimentación^(20)^.

La capacidad de los profesionales para analizar sus experiencias prácticas e identificar áreas de mejora fue un aspecto destacado por una subcategoría. Reflexionar sobre las experiencias pasadas es fundamental para el aprendizaje continuo y la adaptación de las prácticas^(21,22)^. La literatura también sugiere que la capacidad de observar y reflexionar sobre los cambios es importante para implementar mejoras en las prácticas sanitarias^(23)^.

### Feedback y Mejora

La retroalimentación se destacó como un elemento esencial para la autoevaluación y los ajustes en la práctica. La interacción en foros y reuniones presenciales fomentó debates enriquecedores que permitieron a los profesionales identificar lagunas y aplicar estrategias innovadoras. Este hallazgo está en consonancia con la literatura, que posiciona la retroalimentación como una poderosa herramienta para la mejora continua.

La retroalimentación recibida de tutores y colegas se consideró valiosa para realizar ajustes en la práctica profesional. La literatura enfatiza que el feedback constructivo es un componente crítico del aprendizaje reflexivo, ya que permite a los profesionales identificar áreas de mejora y desarrollar estrategias para abordarlas^(24)^. La retroalimentación es una de las herramientas más poderosas para la mejora continua, ya que ofrece información valiosa sobre las prácticas actuales y las posibles mejoras^(25)^.

La planificación de la mejora y la autoevaluación han surgido como componentes esenciales del desarrollo profesional continuo. Estudios recientes afirman que la autoevaluación ayuda a los profesionales a reconocer sus propias competencias y limitaciones, promoviendo un ciclo de mejora continua^(26)^. La autoevaluación es fundamental para el desarrollo de una práctica reflexiva, en la que los profesionales evalúan y ajustan constantemente sus enfoques para satisfacer mejor las necesidades de los pacientes^(21)^.

El intercambio de experiencias en foros y reuniones presenciales permitió la reflexión colectiva, esencial para ampliar la comprensión de las prácticas asistenciales. Este hallazgo se ve corroborado por estudios que demuestran que la colaboración entre profesionales de diferentes áreas de especialización enriquece la práctica y promueve un enfoque más holístico de la asistencia. El aprendizaje situado en comunidades de práctica ofrece un rico intercambio de conocimientos y experiencias que es fundamental para la mejora continua^(27)^.

### Revisión Crítica y Planificación

Esta subcategoría hace hincapié en la importancia de revisar las estrategias y planificar las acciones en función de los retos prácticos. Herramientas de colaboración como los foros y los debates cara a cara han facilitado la profundización del conocimiento y la innovación en las prácticas asistenciales.

En la subcategoría «Revisión crítica y planificación», los graduados destacaron la importancia de revisar las estrategias y reflexionar sobre las dificultades a las que se enfrentan en la práctica. La literatura sugiere que revisar críticamente las estrategias permite a los profesionales ajustar sus enfoques basándose en la evidencia y en las mejores prácticas^(28)^. Esta práctica es esencial para garantizar que la atención prestada sea siempre la más eficaz y se base en las mejores pruebas disponibles.

La revisión de materiales y la discusión de técnicas fueron métodos eficaces para profundizar en los conocimientos y mejorar las prácticas profesionales. El aprendizaje continuo y la revisión de los materiales didácticos son esenciales para mantenerse al día de las mejores prácticas y de las últimas evidencias^(21)^. La revisión crítica y la discusión de técnicas son fundamentales para la innovación y la mejora de las prácticas sanitarias^(23)^.

La revisión de los procesos se ha identificado como un paso importante para consolidar el aprendizaje y garantizar la mejora continua de las prácticas sanitarias. Este hallazgo se ve respaldado por estudios que demuestran que la revisión periódica de los procesos permite a los profesionales identificar ineficiencias e implementar mejoras^(1)^. Además, la revisión sistemática de los procesos es esencial para implantar prácticas basadas en la evidencia y adaptarse a las necesidades cambiantes de los pacientes^(29)^.

### Limitaciones y Perspectivas Futuras

Este estudio tiene limitaciones relacionadas con la generalizabilidad de los resultados, debido al enfoque exclusivo en graduados de un curso específico. Futuras investigaciones podrían explorar el impacto de la formación reflexiva longitudinalmente y comparar diferentes enfoques pedagógicos, profundizando en la comprensión de las competencias reflexivas en la asistencia sanitaria.

Aunque este estudio ha proporcionado resultados valiosos sobre el desarrollo de competencias reflexivas y críticas por parte de los profesionales sanitarios, es importante reconocer algunas limitaciones. En primer lugar, la muestra estuvo compuesta exclusivamente por graduados de un curso específico de especialización en enfermedades crónicas no transmisibles, lo que puede limitar la generalizabilidad de los resultados a otras áreas de especialización o contextos educativos. En segundo lugar, la naturaleza cualitativa del estudio, basado en entrevistas semiestructuradas, puede estar sujeta a sesgos en los autoinformes y en la interpretación de los datos, aunque hayan sido validados por los propios participantes en la investigación.

Además, este estudio se centró predominantemente en la dimensión de Observación Reflexiva del Ciclo de Aprendizaje Experiencial de Kolb, sin explorar otras dimensiones del ciclo, como la experiencia concreta, la experimentación activa y la conceptualización abstracta, que también son importantes para el pleno desarrollo de las competencias profesionales.

Por último, futuras investigaciones podrían explorar longitudinalmente el impacto de las competencias desarrolladas a lo largo del tiempo, evaluando cómo influye la formación reflexiva en la práctica profesional y en la calidad de la asistencia a largo plazo. Los estudios comparativos entre diferentes programas de formación y métodos pedagógicos también serían valiosos para identificar los enfoques más eficaces para desarrollar competencias críticas y reflexivas entre los profesionales sanitarios.

## CONCLUSIÓN

Este estudio subraya la importancia de la problematización, la reflexión crítica y la OR en la formación de los profesionales sanitarios, en particular en el contexto de la gestión de las enfermedades crónicas no transmisibles, mediante metodologías que favorecen el análisis en profundidad de las prácticas cotidianas, como los diarios de campo, los foros de discusión y las reuniones sincrónicas. Fue posible identificar mejoras significativas en los enfoques asistenciales. Estas herramientas educativas no sólo facilitan el aprendizaje colaborativo, sino que promueven una cultura de autoevaluación y mejora continua entre los profesionales.

La educación permanente en salud, a través de la problematización de situaciones cotidianas y de metodologías grupales, motiva a los profesionales sanitarios a reflexionar sobre sus prácticas y a resignificar sus hallazgos. El intercambio de experiencias e ideas entre profesionales de diferentes contextos contribuye a un análisis más rico y detallado de las experiencias, promoviendo la implementación de cambios efectivos.

La integración de la TAE de Kolb en el currículo de especialización ha demostrado ser un modelo eficaz para el desarrollo de habilidades críticas y reflexivas. Este enfoque abre puertasst para que los profesionales de la salud adapten sus prácticas a las realidades específicas de sus territorios, mejorando la calidad de la atención y respondiendo con mayor precisión a las necesidades individuales y colectivas.

Sin embargo, el estudio también revela retos persistentes, como la sobrecarga de trabajo y la escasez de recursos, que repercuten en la aplicación de estrategias preventivas y de atención sanitaria integral. Estos obstáculos ponen de manifiesto la necesidad de políticas públicas más sólidas y de seguir invirtiendo en formación y apoyo a los profesionales sanitarios.
